# X-Ray Pulsar-Based Navigation Considering Spacecraft Orbital Motion and Systematic Biases

**DOI:** 10.3390/s19081877

**Published:** 2019-04-19

**Authors:** Mengfan Xue, Yifang Shi, Yunfei Guo, Na Huang, Dongliang Peng, Ji’an Luo, Han Shentu, Zhikun Chen

**Affiliations:** School of Automation, Hangzhou Dianzi University, Xiasha Higher Education Zone, 2nd Street, Hangzhou 310018, China; xuemf@hdu.edu.cn (M.X.); syf2008@hdu.edu.cn (Y.S.); gyf@hdu.edu.cn (Y.G.); dlpeng@hdu.edu.cn (D.P.); luojian@hdu.edu.cn (J.L.); hanshentu@hdu.edu.cn (H.S.); czk@hdu.edu.cn (Z.C.)

**Keywords:** X-ray pulsar-based navigation, Doppler effect, systematic biases, DPP, photon-level simulation

## Abstract

The accuracy of X-ray pulsar-based navigation is greatly affected by the Doppler effect caused by the spacecraft orbital motion and the systematic biases introduced by the pulsar directional error, spacecraft-borne clock error, etc. In this paper, an innovative navigation method simultaneously employing the pulse phase (PP), the difference of two neighbor PPs (DPP) and the Doppler frequency (DF) of X-ray pulsars as measurements is proposed to solve this problem. With the aid of the spacecraft orbital dynamics, a single pair of PP and DF relative to the spacecraft’s state estimation error can be estimated by using the joint probability density function of the arrival photon timestamps as the likelihood function. The systematic biases involved to the PP is proved to be nearly invariant over two adjacent navigation periods and the major part of it is eliminated in the DPP; therefore, the DPP is also exploited as additional navigation measurement to weaken the impact of systematic biases on navigation accuracy. Results of photon-level simulations show that the navigation accuracy of the proposed method is remarkably better than that of the method only using PP, the method using both PP and DF and the method using both PP and DPP for Earth orbit.

## 1. Introduction

Pulsars are strongly magnetized and rapidly rotating neutron stars emitting signals that are unique and highly periodical [1,2]. Along with their spatial diversity about the galactic disk, pulsars have been suggested as a natural lighthouse for spacecraft navigation. Among all pulsars, X-ray pulsar are more suitable for spacecraft navigation due to the much smaller detector size required compared to that for radio or optical sources [3]. X-ray pulsar-based navigation (XPNAV) has been suggested as a potential approach for autonomous spacecraft navigation [4,5,6]. Unlike some current applications for spacecraft navigation, such as Deep Space Network and Global Navigation Satellite System, which suffer from low performance outside their effective coverage and rely extensively on ground-based operations, the XPNAV has the same accuracy throughout the solar system and has much more autonomy [7,8]. It can also be used to augment the current navigation systems to improve their performance by introducing pulsar measurements [9,10].

In XPNAV, a key task is the estimation of pulse phase (PP), which requires conversion of the measured photon time-series to the coordinate time at the Solar System Barycenter (SSB) using the initial position estimate of the spacecraft and various ephemeris parameters [6,11,12]. Many studies of XPNAV, such as [13,14], have assumed the spacecraft position is invariable within a filtering period and have no consideration of the ephemeris errors and the satellite-borne clock error. Reference [13] presents an XPNAV augmentation method which utilizes both pulsar observation and X-ray ranging observation for navigation filtering. Reference [14] shows how the problem of pulsar phase estimation can be recast as a cyclic shift parameter estimation problem under multinomial distributed observations, whose maximum likelihood solution can be implemented by means of a fast, Discrete Fourier Transform based procedure. However, the un-modeled effects caused by the orbital motion of the spacecraft would severely degrade the PP estimation accuracy and therefore lower the navigation performance; furthermore, ephemeris errors and spacecraft-borne clock error can introduce systematic biases that cannot be effectively suppressed by navigation filtering [9,15,16]. 

To eliminate the spacecraft orbital motion effect, some researchers proposed to use the digital phase-locked loop to track the Doppler frequency (DF) over a sufficiently short interval. This strategy suffers from relatively poor accuracy due to the limited observation duration and strong background noise [17,18]. Some researchers proposed to estimate the spacecraft’s velocity by minimizing the distortion of the pulse profile and have achieved inspiring estimation accuracy [19]. Reference [20] formulated an approximation to the phase evolution at the spacecraft by utilizing the orbit dynamic model and then parameterized a correction to this approximation. This method uses both the PP and the DF as navigation measurements and outperforms the method which only use the PP measurement. Reference [21] proposes a Doppler compensation method for the interplanetary trajectory cruise phase under the assumption that the acceleration of the explorer is constant during a pulsar signal observation period. Reference [22] introduces a pulsar phase and Doppler frequency estimation method using on-orbit epoch folding, in which a framework called on-orbit epoch folding is provided, consisting of removal of vehicle orbital motion effect, search for the period of converted photon time of arrivals, and estimation of the initial phase.

All above mentioned methods haven’t consider the systematic biases. Reference [23] proposes a time-differenced technique to get rid of the systematic biases for spacecraft in constellations. Since this technique requires the pulse time-of-arrival (TOA) measurements of several spacecraft simultaneously, it cannot be applied to a single spacecraft. Reference [24] handles the systematic biases by including them as a part of the state vector, but since it establishes the systematic biases as constant, this method is only appropriate to treat the nearly invariant biases. Assuming the spacecraft is stationary within a filtering period, [9] utilizes the time-differenced pulse TOAs as additional measurements to augment the traditional star angle based navigation and has achieved a good robustness to the spacecraft-borne clock error and the pulsar directional error.

In this paper, inspired by the study in [9], we develop an innovative X-ray pulsar-based navigation method considering both the spacecraft orbital motion effect and the systematic biases introduced by the ephemeris errors and the satellite-borne clock error, of which the PP, the difference of two neighbor PPs (DPP) and the DF of X-ray pulsars observed at the spacecraft are utilized as the navigation measurements. With the aid of the orbit dynamic model, a single pair of PP and DF relative to the spacecraft’s state estimation error can be extracted by recognizing the joint probability density function (pdf) of the arrival photon timestamps as the likelihood function. The systematic biases involved to PP and DF by the ephemeris errors and the satellite-borne clock error are discussed; based on this, the DPP is also introduced as navigation measurement to weaken the impact of systematic biases on navigation accuracy since the major part of systematic biases is eliminated in the DPP. In addition, since the previous PP estimate and the previous state are needed to formulate the DPP measurement equation, which fails to meet the requirements of the standard Kalman filter, a modified unscented Kalman filter (UKF) is deduced to fuse the different measurements; however, it is worthwhile to notice that this modification strategy is flexible, any existing nonlinear estimation approaches can be merged into it. For example, particle filtering is capable of attaining asymptotically optimal estimation of the state of a nonlinear and/or non-Gaussian discrete-time dynamical system [25,26], which is exactly a good choice for the XPNAV. The modification strategy can also be extended to the various state of the art particle filters [27,28].

The rest of the paper is organized as follows. In Section 2, mathematical models of X-ray pulsar signals observed at the spacecraft are deduced based on the ones established at the SSB. In Section 3, the models of the proposed navigation system are developed. The PP, DF and DPP measurements are detailed in this section. Section 4 gives the algorithms needed to estimate the spacecraft’s state utilizing the detected photon timestamps, including a maximum likelihood (ML) estimator used to estimate the navigation measurements and a modified unscented Kalman filter used to fuse the different measurements. A series of photon-level simulations are performed in Section 5 to evaluate the performance of this new navigation method. Finally, Section 6 concludes the study.

## 2. Signal Models of X-Ray Pulsars

The fundamental data provided to the XPNAV system are the observed TOAs of X-ray photons from the pulsar sources and cosmic X-ray background. The foregone photon arrival model of X-ray pulsars is established at a hypothetical reference location usually chosen as the SSB. The photon arrival model at the spacecraft can be obtained by transferring the one at the SSB to the spacecraft according to the spacecraft’s position and velocity.

### 2.1. Photon Arrival Rate Function at the SSB

Assume there exists an X-ray detector at the SSB. Let $\left(t_{0},t_{f}\right)$ be the observation interval. Let $t_{i}$ be the TOA of the *i*-th photon, and the set $\left\{t_{0},t_{1},\cdots ,t_{K}\right\}$, denoted by ${\left\{t_{i}\right\}}_{i=1}^{K}$, be a random sequence in increasing order. The number of the detected photons, K, is also a random variable. Let $t_{0}=\;0$, $N_{0}=\;0$, and $N_{t}$ be the number of detected photons in the interval (0,t). The detected X-ray photon event timestamps $\left\{N_{t},t>0\right\}$ can be modeled as the arrival times of a Non-Homogeneous Poisson Process (NHPP) with a time varying rate function $\lambda_{\mathrm{SSB}}(t)\geq 0$ [2]. In other words, $\left\{N_{t},t>0\right\}$ satisfies the following conditions [2]:

(1) The probability of detecting one photon in a time interval Δt is $\lambda_{\mathrm{SSB}}(t)\Delta t$ when Δt→0.
(2) The probability of detecting more than one photon in Δt is 0 when Δt→0.
(3) Nonoverlapping increments are independent.

For a fixed time interval (s,e), the number of arrival photons $N_{s,e}$ is a Poisson random variable with parameter $\int_{s}^{e}\lambda_{\mathrm{SSB}}(t)dt$. Its distribution law is:

(1) $$P(N_{s,e}=k)=\frac{{\left(\int_{s}^{e}\lambda_{\mathrm{SSB}}(t)dt\right)}^{k}\exp\left(-\int_{s}^{e}\lambda_{\mathrm{SSB}}(t)dt\right)}{k!}$$

The rate function $\lambda_{\mathrm{SSB}}(t)$ align includes all the arriving photons from the X-ray pulsar and the background. It has the form:

(2) $$\lambda_{\mathrm{SSB}}(t)=\beta_{\mathrm{SSB}}+\alpha_{\mathrm{SSB}}h(\phi_{\mathrm{SSB}}(t))\;(\mathrm{ph}/s)$$

where $\alpha_{\mathrm{SSB}}\geq 0$ and $\beta_{\mathrm{SSB}}\geq 0$ are respectively the known effective source and background arrival rates in unit of photons per second, and *h* is the normalized pulsar profile, which is unique to a particular pulsar, defines the characteristic shape of the rate function, and is a continuous and nonnegative real function, periodic with period 1 (h(ϕ+n)=h(ϕ) for ∀n∈Z) and with unit area $\int_{0}^{1}h(\phi )d\phi =1$ [2]. $\phi_{\mathrm{SSB}}(t)$ is the observed phase at the SSB with respect to the coordinate time t seen at the SSB. It is expressed as the Taylor series:

(3) $$\phi_{\mathrm{SSB}}(t)=\phi_{0}+f(t-t_{0})+\frac{\dot{f}}{2}{\left(t-t_{0}\right)}^{2}+\frac{\ddot{f}}{6}{\left(t-t_{0}\right)}^{3}$$

where f,$\dot{f}$ and $\ddot{f}$ are respectively the source frequency and its first and second derivatives, and $\phi_{0}$ the initial phase at a reference time $t_{0}$.

### 2.2. Photon Arrival Rate Function at the Spacecraft

Let r(t) and v(t) respectively denote the spacecraft’s coordinate and velocity in the SSB coordinate frame at the spacecraft proper time t, and n is the directional vector from the SSB to the pulsar. The photon arrival rate function at the spacecraft λ(t) can be written as:

(4) $$\lambda (t)=(1+(n\cdot v(t))/c)(\beta_{\mathrm{SSB}}+\alpha_{\mathrm{SSB}}h(\phi_{\mathrm{SSB}}(t+\tau (t))))$$

with τ(t) the offset of proper time a photon arrives at the spacecraft compared to the arrival SSB-based coordinate time of the same photon at the SSB. For Earth-orbiting spacecraft, assuming that the spacecraft proper time is chosen as the Terrestrial Time (TT), τ(t) can be expressed as [29,30]:

(5) $$\begin{array}{ll} \tau (t) & =\frac{n\cdot r(t)}{c}+\frac{1}{2cD}\left[{\left(n\cdot r(t)\right)}^{2}-{\left|r(t)\right|}^{2}+2(n\cdot r(t))(n\cdot b)-2(b^{T}\cdot r(t))\right]\\ & -\frac{2\mu_{s}}{c^{3}}\ln(1+\frac{\left(n+b^{T})\cdot \left(r(t)+b\right)\right)}{\left|r(t)+b\right|\left|n+b^{T}\right|})+\frac{1}{c^{2}}{\left(\nu (t)-\nu_{E/\mathrm{SSB}}(t)\right)}^{T}\cdot v_{E/\mathrm{SSB}}(t)+P \end{array}$$

where c is the speed of light, *D* is the distance between the Sun and the pulsar, ***b*** is the position of SSB with respect to the Sun, $\mu_{s}$ is the gravitational constant of Sun, $\nu_{E/\mathrm{SSB}}(t)$ is the velocity vector of Earth with respect to the SSB and *P* represents the total periodic correction items. The first term on the right-hand side of Equation (5) is the geometric time delay between the spacecraft and the SSB along the line-of-sight to the pulsar, the second term is due to the effects of parallax, and the third term is the Sun’s Shapiro delay. The last two terms are the Einstein delay, which accomplishes the conversion of spacecraft proper time to SSB-based coordinate time. It is noteworthy that the Sun’s Shapiro delay and the Einstein delay in the Equation (5) is referenced to the ones employed by the Heasoft v6.11.1 software platform provided by NASA [31].

In practical navigation, letting $\tilde{r}(t)$ and $\tilde{v}(t)$ respectively denote the position and velocity of the spacecraft predicted by the orbit dynamics and letting Δr(t) and Δν(t) respectively denote the corresponding position and velocity errors, the accurate position and velocity of spacecraft can be expressed as:

(6) $$r(t)=\tilde{r}(t)+\Delta r(t)$$

(7) $$\nu (t)=\tilde{v}(t)+\Delta \nu (t)$$

Substituting Equations (6)–(7) into Equation (4) and regarding the pulsar frequency as invariant within a time duration of $\frac{n\cdot \Delta r(t)}{c}$, we can expand the estimated phase model at the spacecraft $\phi_{\mathrm{SSB}}(t+\tau (t))$ as [20]:

(8) $$\begin{array}{l} \phi_{\mathrm{SSB}}(t+\tau (t))=\phi_{\mathrm{SSB}}(t+\frac{n\cdot \tilde{r}(t)}{c}+\frac{n\cdot \Delta r(t)}{c}+p(\tilde{r}(t)+\Delta r(t))+s(\tilde{r}(t)+\Delta r(t))+e(\tilde{v}(t)+\Delta \nu (t)))\\ =\tilde{\phi }(t)+{\dot{\phi }}_{\mathrm{SSB}}(t+\frac{n\cdot \tilde{r}(t)}{c}+p(\tilde{r}(t))+s(\tilde{r}(t))+e(\tilde{v}(t)))\frac{n\cdot \Delta r(t)}{c} \end{array}$$

in which:

(9) $$\tilde{\phi }(t)=\phi_{\mathrm{SSB}}(t+\frac{n\cdot \tilde{r}(t)}{c}+p(\tilde{r}(t))+s(\tilde{r}(t))+e(\tilde{v}(t)))$$

is the pulsar phase model at the spacecraft derived by the spacecraft state with errors, $p(\tilde{r}(t)+\Delta r(t))$, $s(\tilde{r}(t)+\Delta r(t))$ and $e(\tilde{v}(t)+\Delta \nu (t))$ respectively represent the parallax delay, Sun’s Shapiro delay and Einstein delay. The effects of spacecraft state error on them are extremely small and therefore they have been neglected.

## 3. Dynamic and Measurement Models of Proposed Navigation Method

The spacecraft’s position x(t) and velocity ε(t) in the J2000.0 Earth Centered Inertial (ECI) coordinate system are selected as the state vector to be estimated. Letting $\left[t_{s},t_{e}\right]$ denote the time interval of a filtering period, the navigation measurements are the estimated phase $\hat{\phi }(t_{e})$ and Doppler frequency $\dot{\hat{\phi }}(t_{e})$ at the time $t_{e}$ as seen at the spacecraft, and the difference of $\hat{\phi }(t_{e})$ and the estimated phase of the previous filtering period, $\hat{\phi }(t_{s})$. In what follows, the Dynamic model and measurement models of the proposed navigation method are formulated in detail.

### 3.1. Dynamics Model

In the Earth-centered inertial coordinate system, the dynamic model of earth-orbit spacecraft can be expressed as:

(10) $$\left[\begin{array}{c} \dot{x}\\ \dot{\varepsilon } \end{array}\right]=\left[\begin{array}{c} \varepsilon \\ a \end{array}\right]+\left[\begin{array}{c} w_{\varepsilon }\\ w_{a} \end{array}\right]$$

where $X={\left[\begin{array}{cc} x^{T} & \varepsilon^{T} \end{array}\right]}^{T}$ is the state vector of spacecraft with respect to the Earth,$W={\left[\begin{array}{cc} w_{\varepsilon }^{T} & w_{a}^{T} \end{array}\right]}^{T}$ is the process noise which can be characterized as a zero-mean Gaussian white noise process, and the acceleration of spacecraft a can be written as:

(11) $$a=a_{\mathrm{earth}}+a_{\mathrm{sun}}+a_{\mathrm{moon}}+a_{J2}$$

considering the two-body effect of Earth $a_{\mathrm{earth}}$, the third-body effects from the Sun and the Moon, respectively denoted by $a_{\mathrm{sun}}$ and $a_{\mathrm{moon}}$, and the Earth non-spherical perturbation acceleration $a_{J2}$ [10].

### 3.2. Measurement Models

#### 3.2.1. PP and DF Measurements

Within a filtering period seen at the spacecraft, regarding Δν(t) and ${\dot{\phi }}_{\mathrm{SSB}}(t+\frac{n\cdot \tilde{r}(t)}{c}+p(\tilde{r}(t))+s(\tilde{r}(t))+e(\tilde{v}(t)))$ as invariant and equal to the one at the time $t_{s}$, Equation (8) can be further simplified as:

(12) $$\phi_{\mathrm{SSB}}(t+\tau (t))=\tilde{\phi }(t)+\Delta p+\Delta f(t-t_{s}),$$

where $\Delta p=f_{s}\frac{n\cdot \Delta r(t_{s})}{c}$, $\Delta f=f_{s}\frac{n\cdot \Delta v(t_{s})}{c}$ and:

(13) $$f_{s}={\dot{\phi }}_{\mathrm{SSB}}(t_{s}+\frac{n\cdot \tilde{r}(t_{s})}{c}+p(\tilde{r}(t_{s}))+s(\tilde{r}(t_{s}))+e(\tilde{v}(t_{s}))).$$

Δp and Δf are respectively the pulse phase delay and Doppler frequency offset which can be estimated from the detected photon event timestamps. The PP measurement equation is given as:

(14) $$\begin{array}{ll} \tilde{\phi }(t_{e})+\Delta \hat{p}\;+\Delta \hat{f}\cdot T_{\mathrm{obs}} & =\phi_{\mathrm{SSB}}(t_{e}+\frac{n\cdot (x(t_{e})+r_{E/\mathrm{SSB}}(t_{e}))}{c}+p(x(t_{e})+r_{E/\mathrm{SSB}}(t_{e}))\\ & +s(x(t_{e})+r_{E/\mathrm{SSB}}(t_{e}))+e(\varepsilon (t_{e})+\nu_{E/\mathrm{SSB}}(t_{e})))+u_{p}(t_{e}) \end{array},$$

with $r_{E/\mathrm{SSB}}(t)$ being the Earth position vector relative to the SSB, $T_{\mathrm{obs}}=\;t_{e}-t_{s}$ the integration interval of navigation filter and $u_{p}(t_{e})$ the phase noise. $\hat{\phi }(t_{e})\;=\;\tilde{\phi }(t_{e})+\Delta \hat{p}\;\;+\;\Delta \hat{f}\cdot T_{\mathrm{obs}}$ is the PP measurement, where $\Delta \hat{p}\;$ and $\Delta \hat{f}$ are respectively the estimated pulse phase delay and Doppler frequency offset.

The DF measurement equation is obtained by differentiating Equation (14) and is written as:

(15) $$\begin{array}{ll} \dot{\tilde{\phi }}(t_{e})+\Delta \hat{f} & ={\dot{\phi }}_{\mathrm{SSB}}(t_{e}+\frac{n\cdot (x(t_{e})+r_{E/\mathrm{SSB}}(t_{e}))}{c}+p(x(t_{e})+r_{E/\mathrm{SSB}}(t_{e}))+s(x(t_{e})+r_{E/\mathrm{SSB}}(t_{e}))\\ & +e(\varepsilon (t_{e})+\nu_{E/\mathrm{SSB}}(t_{e})))\left(1+\frac{n\cdot (\varepsilon (t_{e})+v_{E/\mathrm{SSB}}(t_{e}))}{c}\right)+u_{f}(t_{e}) \end{array}$$

where $u_{f}(t_{e})$ is the frequency noise. The term $\dot{\hat{\phi }}(t_{e})\;=\;\dot{\tilde{\phi }}(t_{e})+\Delta \hat{f}=f_{s}(1+n\cdot \tilde{\nu }(t_{e})/c)\;+\;\Delta \hat{f}$ is the DF measurement, in which the differentials of $p(\tilde{r}(t))$, $s(\tilde{r}(t))$ and $e(\tilde{v}(t))$ are neglected since they are extremely small compared to 1 and $n\cdot \tilde{\nu }(t_{e})/c$.

#### 3.2.2. DPP Measurement

Taking the pulsar directional error, pulsar distance error and spacecraft-borne clock error into consideration, the PP measurement equation can be rewritten as:

(16) $$\begin{array}{l} \hat{\phi }(t_{e})\approx \phi_{\mathrm{SSB}}(t_{e}+\frac{\tilde{n}\cdot \tilde{r}(t_{e})}{c}+\frac{1}{2c\tilde{D}}\left[{\left(\tilde{n}\cdot \tilde{r}(t_{e})\right)}^{2}-{\left|\tilde{r}(t_{e})\right|}^{2}+2(\tilde{n}\cdot \tilde{r}(t_{e}))(\tilde{n}\cdot b)-2(b^{T}\cdot \tilde{r}(t_{e}))\right]\\ -\frac{2\mu_{s}}{c^{3}}\ln(1+\frac{\left(\tilde{n}+b^{T})\cdot \left(\tilde{r}(t_{e})+b\right)\right)}{\left|\tilde{r}(t_{e})+b\right|\left|\tilde{n}+b^{T}\right|})+e(\tilde{v}(t_{e})))+E(\Delta n)+E(\Delta D)+E(\Delta t) \end{array}$$

where $\tilde{n}$ is the measured pulsar direction vector, $\tilde{D}$ is the measured distance between the Sun and the pulsar, Δn the pulsar direction error, ΔD the pulsar distance error, Δt the spacecraft-borne clock error. E(Δn), E(ΔD) and E(Δt) are the corresponding systematic biases caused by Δn, ΔD and Δt, respectively. E(Δn) and E(ΔD) can respectively be approximated by the first order Taylor series:

(17) E(Δn)≈fs(Δn⋅r˜(te)c+1cD˜[(n˜⋅r˜(te))(Δn⋅r˜(te))+(n˜⋅r˜(te))(Δn⋅b)+(Δn⋅r˜(te))(n˜⋅b)]−2μsc3Δn[r˜(te)+b−((n˜+bT)⋅(r˜(te)+b)|n˜+bT|)(d|n˜+bT|dn˜)]|r˜(te)+b||n˜+bT|+(n˜+bT)⋅(r˜(te)+b)≈fsΔn⋅r˜(te)c

and:

(18) $$E(\Delta D)\approx -f_{s}\frac{\Delta D}{2c{\tilde{D}}^{2}}\left[{\left(\tilde{n}\cdot \tilde{r}(t_{e})\right)}^{2}-{\left|\tilde{r}(t_{e})\right|}^{2}+2(\tilde{n}\cdot \tilde{r}(t_{e}))(\tilde{n}\cdot b)-2(b^{T}\cdot \tilde{r}(t_{e}))\right].$$

The derivation of E(Δn) satisfies:

(19) $$\dot{E}(\Delta n)\approx f_{s}\frac{\Delta n\cdot \tilde{v}(t_{e})}{c}\leq \frac{f_{s}}{c}\cdot \left\|\Delta n\right\|\cdot \left\|\tilde{v}(t_{e})\right\|\leq \frac{f_{s}}{c}\cdot v_{\max}\cdot \left\|\Delta n\right\|$$

where $v_{\max}$ is the spacecraft’s maximum velocity in the SSB coordinate frame. Taking the PSR B1821-24 for example and assuming the maximum velocity of the spacecraft $v_{\max}$ is 30 km/s, $\dot{E}(\Delta n)$ is smaller than $2\;\times \;10^{-9}$, meaning that the increment of E(ΔD) is smaller than $7\;\times \;10^{-6}$ within 1 h, which is obviously ignorable. The derivation of E(ΔD) satisfies:

(20) $$\begin{array}{ll} \dot{E}(\Delta D) & \approx f_{s}\frac{\Delta D}{c{\tilde{D}}^{2}}\left[\tilde{r}{\left(t_{e}\right)}^{T}\tilde{v}(t_{e})-(\tilde{n}\cdot \tilde{r}(t_{e}))(\tilde{n}\cdot \tilde{v}(t_{e}))-(\tilde{n}\cdot \tilde{v}(t_{e}))(\tilde{n}\cdot b)+b^{T}\cdot \tilde{v}(t_{e})\right]\\ & \leq f_{s}\frac{2\Delta D}{c{\tilde{D}}^{2}}\left(\left\|\tilde{r}(t_{e})\right\|+\left\|b\right\|\right)\cdot \left\|\tilde{v}(t_{e})\right\|\approx f_{s}\frac{2\Delta D}{c{\tilde{D}}^{2}}\left(\left\|r_{E/\mathrm{SSB}}(t_{e})\right\|+\left\|b\right\|\right)\cdot \left\|v_{E/\mathrm{SSB}}(t_{e})\right\| \end{array}$$

It can be known based on the JPL DE405 that the maximums of $r_{E/\mathrm{SSB}}(t_{e})$, $v_{E/\mathrm{SSB}}(t_{e})$ and b are respectively $1.5\;\times \;10^{8}$ km, 30 km/s, and $5\;\times \;10^{5}$ km. In addition, based on the study of [32], the minimum and uncertainty of $\tilde{D}$ are respectively assumed as 2 kpc and 30%. Therefore, for PSR B1821-24, $\dot{E}(\Delta D)\;\leq \;1\;\times \;10^{-10}$, suggesting that the increment of E(ΔD) within two adjacent navigation periods can be neglected. Furthermore, it has been studied that the spacecraft-borne clock error increases approximately by 36 ns/h [9], thus the variation of $E(\Delta t)=f_{s}\Delta t$ can also be neglected. 

All the above analysis suggests that the DPP measurement can reduce the major part of E(Δn) E(ΔD) and E(Δt). Following from Equation (14), the DPP measurement equation can be written as:

(21) $$\begin{array}{l} \Delta \hat{\phi }(t_{e})=\hat{\phi }(t_{e})-\hat{\phi }(t_{s})\\ =\phi_{\mathrm{SSB}}(t_{e}+\frac{\tilde{n}\cdot (x(t_{e})+r_{E/\mathrm{SSB}}(t_{e}))}{c}+p(x(t_{e})+r_{E/\mathrm{SSB}}(t_{e}))+s(x(t_{e})+r_{E/\mathrm{SSB}}(t_{e}))+e(\varepsilon (t_{e})+\nu_{E/\mathrm{SSB}}(t_{e})))\\ -\phi_{\mathrm{SSB}}(t_{s}+\frac{\tilde{n}\cdot (x(t_{s})+r_{E/\mathrm{SSB}}(t_{s}))}{c}+p(x(t_{s})+r_{E/\mathrm{SSB}}(t_{s}))+s(x(t_{s})+r_{E/\mathrm{SSB}}(t_{s}))+e(\varepsilon (t_{s})+\nu_{E/\mathrm{SSB}}(t_{s})))\;\\ +u_{p}(t_{e})-u_{p}(t_{s}) \end{array}$$

It is noteworthy that since the DPP measurement can only provide information about the relative position, the PP measurement is necessary to acquire information about the absolute position.

## 4. Measurements Estimation and Fusion Filtering

*In Section 3.2.1, we have modeled* the arrival rate function at the spacecraft with respect to the spacecraft’s foregone state information and state error, seen as Equation (12). It turns out that the derivation of the rate function can be parameterized by a pulse phase delay Δp and a Doppler frequency offset Δf, which are respectively caused by the initial position error and velocity error of the spacecraft and are essential for constructing the navigation measurements. In this section, based on the signal models and navigation system models given in the previous sections, the ML estimator used to estimate Δp and Δf directly from the detected X-ray photon timestamps are presented, and then a modified unscented Kalman filter used to fuse the different measurements to update the spacecraft state are detailed.

### 4.1. ML Estimator

Based on the characteristics of NHPP, the joint pdf of the arrival timestamps ${\left\{t_{i}\right\}}_{i=1}^{K}$ during a filtering period $\left[t_{s},t_{e}\right]$ is derived as [2]:

(22) $$p({\left\{t_{i}\right\}}_{i=1}^{K})=e^{-\int_{t_{s}}^{t_{e}}\lambda (s)ds}\prod_{i=1}^{K}\lambda (t_{i})$$

Utilizing Equation (22) as likelihood function, the unknown parameter (Δp,Δf) can be estimated by:

(23) $$(\Delta \hat{p},\Delta \hat{f})=\arg\underset{\Delta p,\Delta f}{\max}\left\{\begin{array}{l} \sum_{i=1}^{K}\ln\left\{\beta_{\mathrm{SSB}}+\alpha_{\mathrm{SSB}}h\left[\tilde{\phi }(t)+\Delta p+\Delta f(t_{i}-t_{s})\right]\right\}+\\ \sum_{i=1}^{K}\ln(1+\frac{\tilde{v}(t_{i})\cdot n}{c}+\frac{\Delta v(t_{s})\cdot n}{c})-\int_{t_{s}}^{t_{e}}\lambda (s)ds \end{array}\right\}$$

For the second term on the right hand side of Equation (23), since the term $\left(\Delta v(t_{s})\cdot n\right)/c$ dependent of the unknown parameter Δf is much smaller than the other two terms independent of (Δp,Δf), the overall second term can be approximately seen as independent of (Δp,Δf). Furthermore, since the third term $\int_{t_{s}}^{t_{e}}{\bar{\lambda }}_{SC}(s)ds$ is also independent of (Δp,Δf), we end up with the following estimator:

(24) $$(\Delta \hat{p},\Delta \hat{f})=\arg\underset{\Delta p,\Delta f}{\max}\sum_{i=1}^{K}\ln\left\{\beta_{\mathrm{SSB}}+\alpha_{\mathrm{SSB}}h\left[\tilde{\phi }(t)+\Delta p+\Delta f(t_{i}-t_{s})\right]\right\}.$$

The 3*σ* search ranges of Δp and Δf are respectively:

(25) $$range(\Delta p)=\left[-3f_{s}\frac{\left|\sigma (\tilde{r}(t_{s}))\cdot n\right|}{c},3f_{s}\frac{\left|\sigma (\tilde{r}(t_{s}))\cdot n\right|}{c}\right]\;\mathrm{and}$$

(26) $$range(\Delta f)=\left[-3f_{s}\frac{\left|\sigma (\tilde{v}(t_{s}))\cdot n\right|}{c},3f_{s}\frac{\left|\sigma (\tilde{v}(t_{s}))\cdot n\right|}{c}\right],$$

in which $\sigma (\tilde{r}(t_{s}))$ and $\sigma (\tilde{v}(t_{s}))$ are the root mean squared error of $\tilde{r}(t_{s})$ and $\tilde{v}(t_{s})$ respectively. 

When the observation time is sufficiently long, the estimation accuracy of the presented ML estimator can achieve the Cramér-Rao lower bound (CRLB):

(27) $$\mathrm{CRLB}(\Delta p)=\frac{4}{(t_{e}-t_{s})I}\;\mathrm{and}$$

(28) $$\mathrm{CRLB}(\Delta f)=\frac{12}{{\left(t_{e}-t_{s}\right)}^{3}I}$$

with $I=\int_{0}^{1}\frac{{\left(\alpha_{\mathrm{SSB}}\dot{h}(\phi )\right)}^{2}}{\beta_{\mathrm{SSB}}+\alpha_{\mathrm{SSB}}h(\phi )}d\phi$ [20].

**Figure 1:**
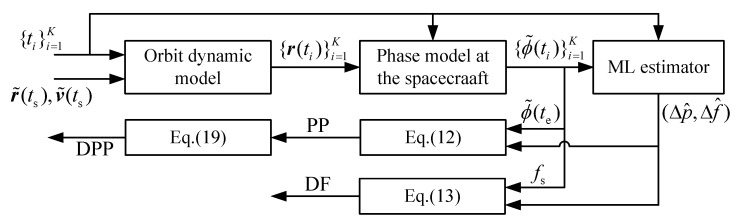
Flowchart of navigation measurements estimation.

After Δp and Δf have been estimated from the observed photon timestamps, the navigation measurements PP, DF and DFF can be obtained according to Equation (14), Equation (15) and Equation (21). Figure 1 shows the overall procedure of extracting navigation measurements from the observed photon timestamps.

### 4.2. Modified Unscented Kalman Filter

Since the orbit dynamic model and the measurement model of the proposed navigation method are both nonlinear, the Kalman filter cannot be used. In the XPNAV, the filtering period is usually several minutes. Under this condition, the EKF, which represents the nonlinear models by a first order Taylor series expansion and neglects the second and higher order terms, cannot perform very well. Since the UKF does not linearize the nonlinear equations, it does not cause the linearization error. For this reason, the UKF is a good choice for the XPNAV [24]. In this section, a modified unscented Kalman filter is deduced to upstate the spacecraft state. In order to reduce the time-correlation of the DPP measurement equation, the previous state which is required in the DPP measurement equation is replaced by the sum of its estimate and the corresponding estimation error, and the mean square error matrix of the DPP measurement noise after the above transformation is derived.

The orbit dynamic model of the proposed navigation system can be rewritten as the following matrix form:

(29) $$X_{k}=f(X_{k-1})+W_{k}.$$

The measurement equations of PP and DF can be expressed in the matrix form:

(30) $$P_{k}=h_{1}(X_{k})+V_{k}\;\mathrm{and}$$

(31) $$F_{k}=h_{2}(X_{k})+U_{k}$$

respectively, in which $P_{k}$ and $F_{k}$ are respectively the PP and DF measurement vectors, and $V_{k}$ and $U_{k}$ are the noise vectors, satisfying $E(V_{k}U_{k}^{T})\;=\;0$, $E(V_{k}W_{k}^{T})\;=\;0$ and $E(U_{k}W_{k}^{T})\;=\;0$. 

Formulating state of the previous epoch $X_{k-1}$ as the sum of its estimate ${\hat{X}}_{k-1}$ and the corresponding estimation error $\Delta X_{k-1}$, the DPP measurement equation can be rewritten as:

(32) $$\begin{array}{ll} D_{k} & =h_{1}(X_{k})-h_{1}(X_{k-1})+V_{k}-V_{k-1}\\ & =h_{1}(X_{k})-h_{1}({\hat{X}}_{k-1}+\Delta X_{k-1})+V_{k}-V_{k-1}\\ & \approx h_{1}(X_{k})-h_{1}({\hat{X}}_{k-1})-H_{k-1}\Delta X_{k-1}+V_{k}-V_{k-1}\\ & =h_{1}(X_{k})-h_{1}({\hat{X}}_{k-1})+\Gamma_{k} \end{array}$$

where $D_{k}$ is the DPP measurement vector:

(33) $$H_{k-1}={\frac{\partial h_{1}(X)}{\partial X}|}_{X={\hat{X}}_{k-1}}$$

and $\;\Gamma_{k}\;=-H_{k-1}\Delta X_{k-1}+V_{k}-V_{k-1}$ is the noise vector, satisfying $E(\Gamma_{k}W_{k}^{T})\;=\;0$ and $E(\Gamma_{k}U_{k}^{T})=0$. 

It can be deduced that:

(34) $$\begin{array}{ll} C_{k} & =\;E(\Gamma_{k}\Gamma_{k}^{T})\\ & =E((-H_{k-1}\Delta X_{k-1}+V_{k}-V_{k-1}\;){\left(-H_{k-1}\Delta X_{k-1}+V_{k}-V_{k-1}\;\right)}^{T})\\ & =H_{k-1}E(\Delta X_{k-1}\Delta X_{k-1}^{T})H_{k-1}^{T}+E(V_{k}V_{k}^{T})+E(V_{k-1}V_{k-1}^{T})+H_{k-1}E(\Delta X_{k-1}V_{k-1}^{T})\\ & =H_{k-1}E(\Delta X_{k-1}\Delta X_{k-1}^{T})H_{k-1}^{T}+Q_{k}+Q_{k-1}-H_{k-1}K_{k-1}R_{k-1} \end{array}$$

where $K_{k-1}$ is the gain matrix and $Q_{k}=E(V_{k}V_{k}^{T})$.

After the above transformations, we can perform the procedures of standard unscented Kalman filter to update the spacecraft’s position and velocity by recognizing Equation (10) as the orbit dynamic model and recognizing Equation (30), Equation (31) and Equation (32) as the measurement equations.

## 5. Photon-Level Simulations and Discussion

In this section, some simulations are carried out to evaluate the performance of the proposed navigation method, using photon event timestamps generated by the simulation method given in [33].

**Figure 2:**
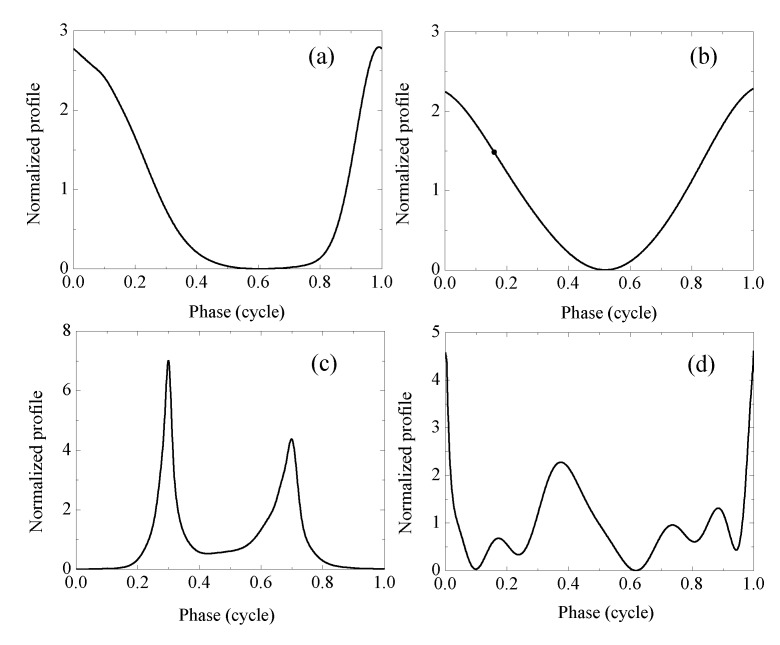
Normalized profiles of pulsar B1509-58 (**a**), B0540-69 (**b**), B0531+21 (**c**) and B0833-45 (**d**).

The pulsars PSR B1509-58, PSR B0540-69, PSR B0531+21 and PSR B0833-45 are selected as the navigation pulsars and their parameters are listed in Table 1. Figure 2 shows the normalized profiles of the selected four pulsars [20]. The initial orbit state is listed in Table 2. The simulation start time is chosen as MJD 52557.1155893. The Earth’s position and velocity is predicted by the ephemeris JPL DE405. The detector area is 1 m^2^ and the filtering period is 120 s. The background radiation flux for PSP B0531+21 is 1.015 ph/cm^2^/s [4]. For the other three pulsars, the background flux is assumed as 0.005 ph/cm^2^/s based on the research of the Naval Research Laboratory. The uncertainty of pulsar distance is set as 30% [32]. The initial clock error and the drift rate of clock frequency are respectively set as 1 μs and 10^−11^ Hz [9].

Considering that the filtering period is longer than the observation time required for the selected four pulsars to achieve the Cramér-Rao lower bound (CRLB) and to account for other un-modeled error sources, the noise levels of $\Delta \hat{p}$ and $\Delta \hat{f}$ are set as two times the square-root of CRLB. The covariance matrix of the process noise is diag$\left[q_{1}^{2},q_{1}^{2},q_{1}^{2},q_{2}^{2},q_{2}^{2},q_{2}^{2}\right]$, where $q_{1}=2\;\times \;10^{-5}\;m$ and $q_{2}=6\;\times \;10^{-4}\;m/s$ [10]. The measurement update period is set as 120 s. The initial position and velocity errors of spacecraft are respectively [100 km, 100 km, 100 km] and [100 m/s, 100 m/s, 200 m/s] and the covariance of the initial state is $\mathrm{diag}[q_{3}^{2},q_{3}^{2},q_{3}^{2},q_{4}^{2},q_{4}^{2},q_{5}^{2}]$, where $q_{3}=100\;\mathrm{km}$, $q_{4}=100\;m/s$ and $q_{5}=200\;m/s$.

First, we compare our proposed method with the traditional method which only uses the PP measurement and the method which uses both the PP and DF measurements. For clarity, Table 3 gives the concepts of different measurements. When performing real-time photon level navigation simulations, the phase of the detected photons must be computed considering the dynamic motion of the spacecraft, otherwise the un-modeled Doppler effects caused by the motion of the spacecraft would result in the distortion of the folded pulse profiles of X-ray pulsars and severely degrade the pulse phase estimation accuracy which directly determines the navigation accuracy. Therefore, focusing on testifying the advantages of incorporating the DF and DPP measurements, in our experiments, we also use Equation (8) given in our article to compute the estimated phase of the detected photon timestamps for the traditional method which only uses the PP measurement. To focus on this purpose, we also use the UKF to update the spacecraft state for the latter two methods.

**Figure 3:**
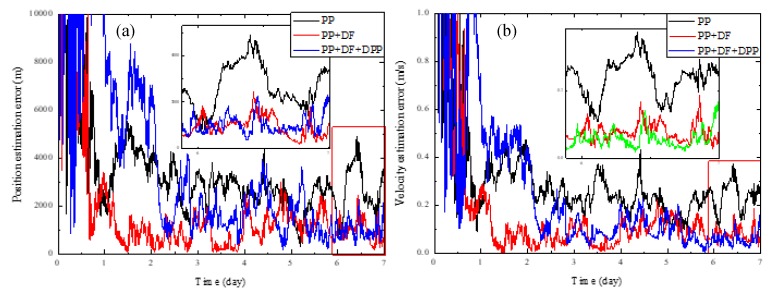
Performance comparison of different navigation measurements without systematic biases. (**a**) Position estimation error; (**b**) Velocity estimation error.

**Figure 4:**
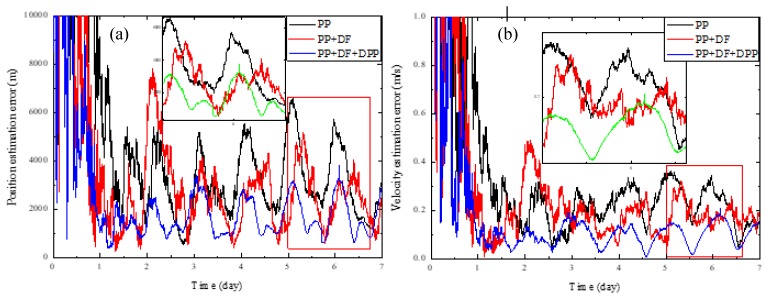
Performance comparison of different navigation measurements with systematic biases. (**a**) Position estimation error; (**b**) Velocity estimation error.

Figure 3 shows the performance comparison of the three methods without systematic biases, and Figure 4 shows the results with systematic biases. For clarity, Table 4 lists the steady-state position and velocity estimation errors corresponding to the results shown in Figure 3 and Figure 4. It can be seen that no matter whether there are systematic biases, the method using the PP and DF measurements both obviously outperform the conventional XPNAV method which only uses the PP measurement, illustrating that the incorporation of the DF measurement in the navigation filter can yield better navigation accuracy. It is because that the modelling and estimation of the DF can eliminate the effect of the spacecraft orbital motion on the PP estimation accuracy to a large extent. 

It can also be observed that our proposed method which incorporates the DPP measurement as well as the PP and DF measurements obviously outperforms the method which only uses the PP measurement no matter whether there are systematic biases. Furthermore, when there are systematic biases, the navigation accuracy of our proposed method is also obviously higher than the one of method using the PP and DF measurements, which verifies that the incorporation of DPP measurement can reduce the effect of the systematic biases on navigation accuracy. However, when there are no systematic biases, it is observed that the performance of our proposed method is slightly worse than the method which uses the PP and DF measurements. We attribute this to the reason that the DPP measurement introduces more noise than useful information in this situation.

**Figure 5:**
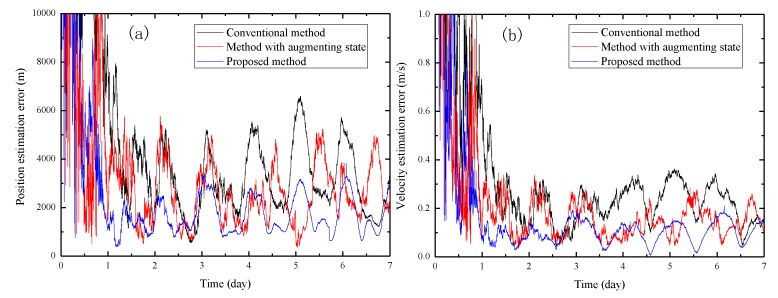
Performance comparison of the proposed method, the method with augmenting state and the conventional method. (**a**) Position estimation error; (**b**) Velocity estimation error.

Figure 5 shows the performance comparison between the proposed method, the method with augmenting state proposed in Ref. [24] and the conventional method which only uses the PP measurement. It can be seen that compared with the conventional method, the other two methods both can reduce the impact of systematic biases. Besides, the proposed method can achieve a more accurate steady state compared to the method with augmenting state. We attribute this to the reason that when focusing on compensating the composite impacts of various systematic biases, our proposed method has a better robustness to measurement noise compared to the method with augmenting state.

The performance of the proposed method is also studied under different initial velocity errors. Table 5 lists the steady-state position estimation errors of our proposed method and the method using PP and DPP measurements under different initial velocity error, with the other parameters being set as same as described above. It can be seen that the position error of the method using only PP and DPP measurements rapidly grows as the initial velocity error increases; on the other hand, position error of our proposed method only slightly grows as the initial velocity error increases and the advantage of our proposed method compared to the method without DF measurements becomes more and more notable. 

**Figure 6:**
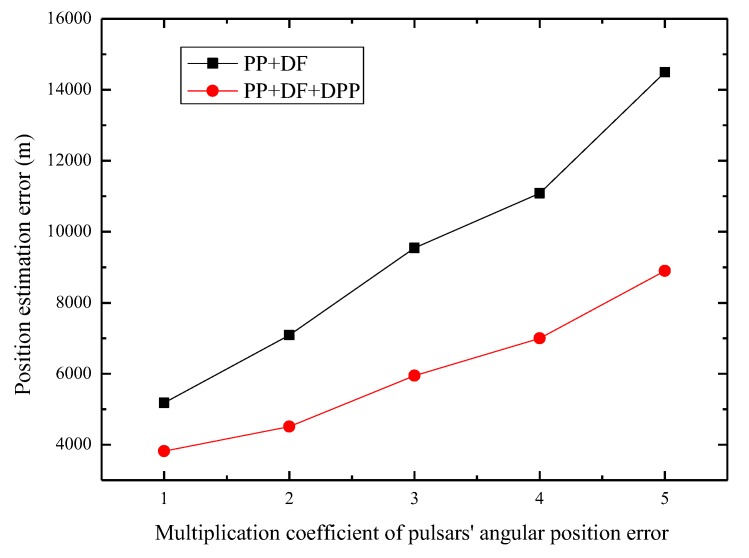
Performance comparison of different angular position errors.

Finally, we investigate the effectiveness of the proposed method under different levels of systematic biases. Due to space limitations, here we only depict the impacts of pulsar directional errors on navigation performance. The impacts of pulsar distance error and spacecraft-borne clock error on navigation performance show the same regularity as that of pulsar directional errors. Figure 6 shows the performance comparison of our proposed method and the method using the PP and DF measurements under different angular position errors. The declination errors and right ascension errors are respectively set as n∗δθ and n∗δφ, of which δθ and δφ are the actual values of the angular position errors listed in Table 1 and n is the multiplication coefficient. We can see that as the pulsar angular position error increases, our proposed method consistently outperforms the method which uses the PP and DF measurements and the advantage becomes more and more notable. It is noteworthy that the position estimation errors of the proposed method also increase as the pulsar angular position error increases. We expect this phenomenon since though the time differential method can eliminate the major part impact of the systematic biases on the DPP measurement, the PP and DF measurements are still affected by the systematic biases, which can lead to a decrease in the navigation performance. 

## 6. Conclusions

This paper develops an innovative navigation method to eliminate the impact of Doppler effect caused by the spacecraft orbital motion and the systematic biases introduced by the pulsar directional error, spacecraft-borne clock error, etc. The method simultaneously employs the PP, the DF and the DPP of X-ray pulsars as navigation measurements. The PP and DF relative to the spacecraft’s state estimation error is estimated by using the joint probability density function of the arrival photon timestamps as the likelihood function. Since the major part of systematic biases introduced by pulsar directional error, pulsar distance error and spacecraft-borne clock error is eliminated in the DPP, the DPP is also employed as navigation measurement to weaken the impact of systematic biases. Results of photon-level simulations testify to the advantages of the proposed method and show that simultaneously incorporating the PP, the DF and the DPP measurements can yield much better navigation accuracy than the method only using PP, the method using both PP and DF and the method using both PP and DPP for Earth orbit, and its advantage becomes more and more notable when the systematic biases increase.

It is noteworthy that the correlation between the PP and DPP measurements will inappropriately bias the filter, which is a shortcoming of the proposed method. If an effective method can be found to eliminate the correlation between the PP and DPP measurements, the navigation accuracy can be further improved, which is our next work. The batch least squares method can also be used to provide the DPP measurement, which is also one of our next work.

## Figures and Tables

**Table 1:** Parameters of navigation pulsars [20,29].

Name	Declination (°)	Right Ascension (°)	D (kpc)	$\alpha_{\mathrm{SSB}}(\mathrm{ph}/{\mathrm{cm}}^{2}/s)$	f (1/s)	$\dot{f}$ (1/s^2^)	Declination Errorδθ (mas)	Right Ascension Errorδφ (mas)
B0833-45	−2.79	263.55	3.9	1.59 × 10^−3^	11.197	−1.56 × 10^−11^	−44	128
B0540-69	−68.6684	85.0465	47.3	5.15 × 10^−3^	19.802	0.000 × 10^+0^	200	40
B1509-58	−58.8642	228.4818	4.3	1.62 × 10^−2^	6.629	−6.74 × 10^−11^	1000	90
B0531+21	22.0145	83.6332	2	1.12 × 10^−1^	29.982	−3.79 × 10^−10^	60	5

**Table 2:** Initial orbit state of the spacecraft.

Orbit State	Value
*x*-axis position	−7385277.8 m
*y*-axis position	34560765.34 m
*z*-axis position	−22339513.83 m
*x*-axis velocity	−1316.58 m/s
*y*-axis velocity	−1702.40 m/s
*z*-axis velocity	−2223.82 m/s

**Table 3:** Concepts of different measurements.

Measurement	Concept
PP	PP Pulse phase $\hat{\phi }(t_{e})$
DF	DF Doppler frequency $\dot{\hat{\phi }}(t_{e})$
DPP	difference of $\hat{\phi }(t_{e})$ and $\hat{\phi }(t_{s})$

**Table 4:** Steady-state position and velocity estimation errors with different navigation measurements.

		PP	PP + DF	PP + DF + DPP
Results without systematic errors	Position error (m)	4906	3010	3297
	Velocity error (m/s)	0.4355	0.2191	0.2214
Results with systematic errors	Position error (m)	6591	5180	3820
	Velocity error (m/s)	0.36	0.32	0.2301

**Table 5:** Steady-state position estimation errors with different initial velocity error.

Initial Velocity Error	Position Error (m) (PP + DPP)	Position Error (m) (PP + DF + DPP)
[60 m/s, 60 m/s, 120 m/s]	4084	3565
[80 m/s, 80 m/s, 160 m/s]	4367	3687
[100 m/s, 100 m/s, 200 m/s]	5298	3824
[120 m/s,120 m/s, 240 m/s]	6496	4078
[140 m/s, 140 m/s, 280 m/s]	7930	4307
[160 m/s, 160 m/s, 320 m/s]	9477	4472

## Appendix A

**Important parameters’ notation and meaning.**

$\lambda_{\mathrm{SSB}}(t)$	time varying photon arrival rate function at the SSB
$\alpha_{\mathrm{SSB}}$	effective source arrival rate
$\beta_{\mathrm{SSB}}$	effective background arrival rate
h(ϕ)	normalized pulsar profile
$\phi_{\mathrm{SSB}}(t)$	observed phase at the SSB with respect to the coordinate time t seen at the SSB
$\phi_{0}$	initial phase at a reference time $t_{0}$ seen at the SSB
r(t)	spacecraft’s coordinate in the SSB coordinate frame at the spacecraft proper time t
v(t)	spacecraft’s velocity in the SSB coordinate frame at the spacecraft proper time t
***n***	directional vector from the SSB to the pulsar
λ(t)	photon arrival rate function at the spacecraft
τ(t)	offset of proper time a photon arrives at the spacecraft compared to the arrival SSB-based coordinate time of the same photon at the SSB
c	speed of light
D	distance between the Sun and the pulsar
b	position of SSB with respect to the Sun
$\mu_{s}$	gravitational constant of Sun
$\nu_{E/\mathrm{SSB}}(t)$	velocity vector of Earth with respect to the SSB
P	total periodic correction items
$\tilde{r}(t)$	position of the spacecraft predicted by the orbit dynamics
$\tilde{v}(t)$	velocity of the spacecraft predicted by the orbit dynamics
Δr(t)	position error
Δν(t)	velocity error
$p(\tilde{r}(t)+\Delta r(t))$	parallax delay corresponding to position $\tilde{r}(t)+\Delta r(t)$
$s(\tilde{r}(t)+\Delta r(t))$	Sun’s Shapiro delay corresponding to position $\tilde{r}(t)+\Delta r(t)$
$e(\tilde{v}(t)+\Delta \nu (t))$	Einstein delay corresponding to velocity $\tilde{v}(t)+\Delta \nu (t)$
$\tilde{\phi }(t)$	pulsar phase model at the spacecraft derived by the spacecraft state with errors
x(t)	spacecraft’s position in the J2000.0 Earth Centered Inertial (ECI) coordinate system
ε(t)	spacecraft’s velocity in the J2000.0 Earth Centered Inertial (ECI) coordinate system
$\left[t_{s},t_{e}\right]$	time interval of a filtering period
$\hat{\phi }(t_{e})$	estimated phase at the time $t_{e}$ as seen at the spacecraft
$\dot{\hat{\phi }}(t_{e})$	estimated Doppler frequency at the time $t_{e}$ as seen at the spacecraft
a	acceleration of spacecraft
X	state vector of spacecraft with respect to the Earth
W	process noise vector
$\begin{array}{cc} w_{\varepsilon }^{} & \end{array}$	velocity noise vector
$\begin{array}{c} w_{a} \end{array}$	acceleration noise vector
Δp	pulse phase delay
Δf	Doppler frequency offset
$r_{E/\mathrm{SSB}}(t)$	Earth position vector relative to the SSB
$u_{p}$	phase noise
$\Delta \hat{p}\;$	estimated pulse phase delay
$\Delta \hat{f}$	estimated Doppler frequency offset
$u_{f}$	frequency noise
$\tilde{n}$	measured pulsar direction vector
Δn	pulsar direction error
$\tilde{D}$	measured distance between the Sun and the pulsar
ΔD	pulsar distance error
E(Δn)	corresponding systematic biases caused by Δn
E(ΔD)	corresponding systematic biases caused by ΔD
E(Δt)	corresponding systematic biases caused by Δt
$v_{\max}$	spacecraft’s maximum velocity in the SSB coordinate frame
$\Delta \hat{\phi }(t_{e})$	the difference of $\hat{\phi }(t_{e})$ and the estimated phase of the previous filtering period, $\hat{\phi }(t_{s})$
P	PP measurement vector of the navigation filter
F	DF measurement vector of the navigation filter
V	PP noise vector of the navigation filter
U	DF noise vector of the navigation filter
D	DPP measurement vector of the navigation filter
Γ	DPP noise vector of the navigation filter
